# An Energy Efficient Compressed Sensing Framework for the Compression of Electroencephalogram Signals

**DOI:** 10.3390/s140101474

**Published:** 2014-01-15

**Authors:** Simon Fauvel, Rabab K. Ward

**Affiliations:** Department of Electrical and Computer Engineering, The University of British Columbia, 2322 Main Mall, Vancouver, BC V6T1Z4, Canada; E-Mail: rababw@ece.ubc.ca

**Keywords:** compressed sensing (CS), electroencephalography (EEG), wireless body sensor network (WBSN), telemedicine, biomedical signal processing

## Abstract

The use of wireless body sensor networks is gaining popularity in monitoring and communicating information about a person's health. In such applications, the amount of data transmitted by the sensor node should be minimized. This is because the energy available in these battery powered sensors is limited. In this paper, we study the wireless transmission of electroencephalogram (EEG) signals. We propose the use of a compressed sensing (CS) framework to efficiently compress these signals at the sensor node. Our framework exploits both the temporal correlation within EEG signals and the spatial correlations amongst the EEG channels. We show that our framework is up to eight times more energy efficient than the typical wavelet compression method in terms of compression and encoding computations and wireless transmission. We also show that for a fixed compression ratio, our method achieves a better reconstruction quality than the CS-based state-of-the art method. We finally demonstrate that our method is robust to measurement noise and to packet loss and that it is applicable to a wide range of EEG signal types.

## 1. Introduction

Healthcare consumes a large part of the gross domestic product of developed countries, and the trend is going upward ([1,2]). Solutions are thus needed to mitigate this issue. One possibility is to enable patients to participate in their own treatment by giving them the technological tools necessary to monitor and communicate their situation to caregivers. With recent advances in signal processing and very-low power wireless communications, wireless body sensor networks (WBSNs) have gained popularity as a potential solution. The use of various sensors located on the patient's body allows WBSNs to measure and communicate different physiological signals (e.g., heart and brain activity) [3].

With respect to electrical brain activity, the electroencephalogram (EEG) signals are recorded using a collection of non-invasive wireless sensors located on a patient's scalp. These signals can then be used to detect different medical conditions, such as epileptic seizures [4]. The detection of seizures through the use of a WBSN offers significant advantages. Because it is a relatively rare occurrence, seizure detection requires constant monitoring for an extended period of time, which is resource-intensive when carried in a health institution. Using an EEG WBSN can circumvent this by providing the patient a way to do the monitoring themselves and then consulting with a physician once the relevant data has been gathered.

Another important application of EEG signals in WBSNs is the use of a brain computer interface (BCI) that can detect the EEG patterns associated with a certain task performed by a patient [5]. The patient could use a mental task (such as attempting to move a finger or some arithmetic task) to operate a wheel chair, switch a light off or communicate with the caregiver. As the signals associated with the mental task will be embedded in the patient's EEG, the EEG signals are analyzed to detect their presence and, thus, operate a device. One of the main components required in the successful use of BCIs in a WBSN context is the development of advanced compression techniques that preserve the relevant information (or features) in the EEG signals [6].

Other common uses of EEG signals include sleep pattern studies and the diagnosis and treatment of strokes, infections (e.g., encephalitis, cerebral abscess), cerebral tumors and diseases (e.g., Alzheimer's) (see e.g., [7–10]). In most of these cases, it is important to have a system that does not hamper the movements of the patient, hence why the cordless nature of WBSNs is valuable.

**Figure 1.**
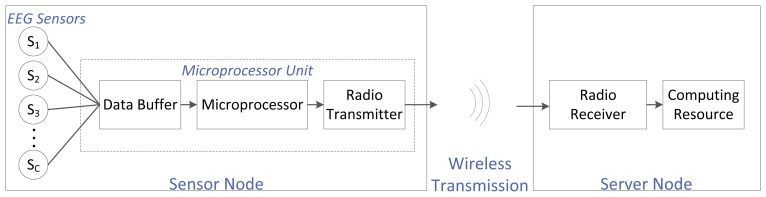
General block diagram for the electroencephalography (EEG) wireless body sensor network (WBSN) system.

For an EEG-based WBSN, the EEG sensors are electrodes placed on a person's head, usually following an international convention (e.g., the international 10–20 system). An EEG sensor is also referred to as the EEG channel. The number of sensors depends on the application: some systems require few electrodes, while others require a few hundred. Every sensor is wired to a single central microprocessor unit that usually has three main components: a buffer (to store the EEG data stream coming from the different EEG channels; this buffer acts as memory), the microprocessor itself (to carry out computations needed before transmission) and a low-power radio (to wirelessly transmit the data). The combination of the EEG sensors and the microprocessor unit is referred to as the sensor node. This sensor node is battery powered. The sensor node transmits the EEG data to the server node wirelessly. The server node is comprised of two main blocks: a low-power radio receiver (to receive the transmitted EEG data) and a computing resource (to carry out any post-transmission computations, storage and any other desired operations). We assume that there is no constraint on the energy supply or the computational power at this server node. The complete system setup is shown in Figure 1.

The energy available in the battery powered sensor node in WBSNs is limited. This energy is needed for: (1) acquiring and digitizing the EEG samples; (2) carrying out the computations at the sensor node; and (3) wirelessly transmitting the data. Under current sensors technology, there is little that can be done to minimize the energy used for acquiring the signals; that is, the raw data must all be acquired and digitized. For computations carried out at the sensor node, energy savings could be realized by using algorithms that have low computational complexity. To minimize the amount of data transmitted, the acquired signals should be compressed before their transmission. A higher compression ratio will minimize the energy required for transmission. In other words, it is crucial to develop compression algorithms that do not require much computational energy.

Traditionally, measurements are collected by the sensors at the Nyquist rate. Then, lossy compression algorithms are directly applied to the raw data, prior to wirelessly transmitting them to the server node. This approach is undesirable for WBSNs, because of its high computational demand (and, thus, high energy consumption).

Recent research has demonstrated the advantages of using compressed sensing (CS) as an alternative compression scheme for physiological signals in the context of WBSNs [11]. CS is a novel paradigm that allows the sampling of the signals at a sub-Nyquist rate. After acquiring the raw data, CS obtains a much smaller number of samples by taking linear projections of the raw data. This is a simple operation, which can be done at a low energy cost. The reconstruction of the data is, however, computationally complex and is allocated to the server node [12]. As no constraints are placed on the computational power and energy resources of the server node, this makes CS appropriate in the context of WBSNs.

The first study that applied CS to EEG compression used the multiple measurements vectors (MMV) approach to compress and reconstruct the signals of the different EEG channels (*i.e.*, all channels are reconstructed simultaneously) [13]. The obtained results were good (high compression ratio for reasonable reconstruction error), but this approach needed EEG signals from repeated trials (asking the patient to repeat the same task many times and recording one EEG channel each time). This setup increases the coherence in the signals (asking someone to carry out the same task is bound to result in EEG signals that are highly coherent). This setting is of limited interest in telemedicine applications, since in these applications, the patient is usually not prompted to act in a certain way or to repeat the same task multiple times.

For telemedicine applications, the first study that addressed the use of CS in EEG signal compression is found in [14,15]. This work focused on surveying existing sparsifying dictionaries and reconstruction algorithms and testing different combinations of these elements to determine which one yielded the best results. The conclusion was that the applicability of single-channel CS for EEG signals depended on the intended application and the tolerable reconstruction error.

More recently, Independent Component Analysis (ICA) was applied as a preprocessing step before using CS for compressing the EEG signals of newborn babies [16]. The compression results obtained were superior to other state-of-the-art methods that do not employ ICA preprocessing. This system, however, consumes much energy at the sensor node and would not be suitable for telemedicine applications. This is because the ICA algorithm is computationally intensive, and such an operation must be carried at the sensor node. The results were later improved, but the computational complexity incurred at the sensor node remained too high for practical systems [17].

In [18], the focus was on developing an efficient hardware architecture for compressed sensing in the context of WBSN. This work demonstrates the potential gains of CS in such a context. However, it has some limitations. The hardware was developed so that it implemented a simple, single-channel version of CS. Limited testing was carried out when it comes to the reconstruction accuracy of the EEG application. Because it is a purely hardware-based architecture, any change in the architecture requires a hardware redesign. Furthermore, it is not possible to compare it against other frameworks unless someone builds the hardware, which is inconvenient.

The above studies resulted in some important questions: (i) What energy savings can be realized by using CS for EEG WBSN applications? (ii) Is it possible to exploit both the temporal correlations (intra-correlations) and the spatial correlations (inter-correlations between channels) to increase the compression performance of CS? (iii) How does CS compare with other state-of-the-art compression algorithms for EEG compression in WBSNs?

In this paper, we propose a novel CS framework that takes advantage of the inherent structure present in EEG signals (both temporal and spatial correlations) to improve the compression performance. To the best of our knowledge, this is also the first time that CS frameworks are compared with other state-of-the-art compression frameworks for EEG compression in WBSNs. It is also the first study where different types of EEG signals representing a variety of applications are used to test the performance of the proposed and existing frameworks, thus providing a more robust answer to the usefulness and validity of the systems.

The paper is organized as follows. Section 2 gives an overview of the theory underlying CS. Section 3 describes our algorithm and briefly introduces the benchmarking algorithms. Section 4 describes the experimental setup used for our experiments. Section 5 presents our results. Finally, Section 6 concludes the paper with suggestions for improvement and future work.

## 2. Compressed Sensing

This section briefly discusses the key theoretical concepts behind compressing sensing: signal sparsity, signal acquisition and reconstruction, measurement incoherence and the extension to compressible signals.

### 2.1. Signal Sparsity

Compressed sensing (CS) exploits the fact that most signals have a sparse representation in some dictionary. Denoting this dictionary by **Ψ***_N_*_×_*_K_* = [*ψ*_1_, *ψ*_2_,…,*ψ_K_*], we can write an original one-dimensional signal, ***f***, of length *N* as:

(1) $$\text{f}=\Psi \text{c}=\sum_{i=1}^{K}c_{i}\psi_{i}$$

When the *K* × 1 vector ***c*** has a large number of zero (or small insignificant) coefficients, ***f*** can be obtained from ***c*** using few dictionary vectors *ψ_i_*. The number of nonzero elements of ***c*** is called the sparsity of ***f***. If there are *S* such elements, it is said that ***c*** is the *S*-sparse representation of ***f*** in dictionary **Ψ**.

### 2.2. Signal Acquisition and Reconstruction

The CS theory implies that instead of acquiring the *N* values of the signal, ***f***, and then compressing it, it is possible to only acquire *M* samples, where *M* is slightly larger than *S* (the “true” information in the signal), but is still much smaller than *N*. This sampling paradigm, referred to as “analog CS”, is the ultimate goal of CS. However, it cannot yet be attained by present day sampling technologies. At present, to represent ***f*** using *M* samples, all the *N* samples are collected, discretized and then subsampled. The subsampling is carried out using *M* linear projections of ***f***. That is, CS subsamples ***f*** using an *M* × *N* sampling matrix Φ, with *M* ≪ *N*, thus creating a measurement vector ***y*** of length *M*:

(2) $${\text{y}}_{M\times 1}=\Phi \text{f}=\Phi \Psi \text{c}$$

This system of equations is largely underdetermined. That is, given a ***y*** vector, there is an infinite number of solutions for ***f*** (or equivalently, ***c***). However, since the signal we wish to recover (***f***) is sparse, the correct solution is often the sparsest solution. This corresponds to solving the following *l*_0_ optimization problem:

(3) $$\underset{c}{\min}\;{\left\|\text{c}\right\|}_{0}\;\text{subject to}\;\text{y}\;=\Phi \Psi \text{c}$$

where the *ℓ*_0_ pseudonorm ‖ · ‖_0_ is the number of non-zero elements in a given vector. Unfortunately, this problem is non-deterministic polynomial-time hard (NP-hard), and as such, it is not tractable. Indeed, solving this problem requires an exhaustive search over all subsets of columns of **Φ**, which is a combinatorial problem [19].

Fortunately, an *ℓ*_1_ optimization problem, a more practical problem due to its convexity, was shown to be equivalent under some conditions. It can be rewritten as follows:

(4) $$\underset{c}{\min}\;{\left\|\text{c}\right\|}_{1}\;\text{subject to}\;\text{y}\;=\Phi \Psi \text{c}$$

This problem can be recast as a linear programming one, for which many practical solvers exist. It has also been shown that perfect recovery can be achieved, even when a small number of measurements (*i.e.*, *M* ≪ *N*) is used [20].

### 2.3. In**c**oherent Measurements

The minimum acceptable value for *M* that allows the perfect reconstruction of the signal, ***f***, is not only linked to the degree of sparsity, *S*, of ***f*** in dictionary Ψ, but also to *μ*, the degree of coherence between **Ψ**; and **Φ**. This coherence measures the largest correlation between any element of **Φ** and **Ψ** and is measured by:

(5) $$\mu (\Phi ,\Psi )=\sqrt{N}\cdot \underset{1\leq l,j\leq N}{\max}\left|\langle \phi_{l},\psi_{j}\rangle \right|$$

The number of measurements *M* is given by:

(6) $$M\geq C\cdot \mu^{2}(\Phi ,\Psi )\cdot S\cdot \log N$$

where ***C*** is a positive constant [12]. Thus, the smaller the coherence, the smaller the value of *M* can be. As such, it is important to select **Ψ** and **Φ**, so that they are maximally incoherent.

Ideally, one should not need to know the sparsifying dictionary, **Ψ**, in order to pick a measurement matrix, **Φ**. Fortunately, some measurement matrices can be shown to be maximally incoherent with any sparsifying dictionary. Random matrices have this property. Indeed, a matrix, **Φ**, generated by independent and identically distributed Gaussian random variables or by Bernoulli random variables would display this property [19]. This means that a random measurement matrix that is properly constructed can allow perfect reconstruction without having any knowledge about the original signal.

### 2.4. Extension to Compressible Signals

CS can further be extended to compressible signals (signals that are not purely sparse in a given dictionary, but whose coefficients, ***c***, decay with a power law when arranged in descending order). This setting is more realistic in practice, as real signals are rarely purely sparse, but are often compressible. This is indeed the case for EEG signals.

Suppose that we are interested in recovering the *P* largest coefficients of the compressible signal, ***f*** (where *P* ≪ *N*). That is, we want to recover the indices, as well as the magnitudes of the *P* largest values of ***c***. Suppose that the number of collected measurements (*M*) is equal to *P*, *i.e.*, only *P* ≪ *N* random projections of the signal are collected. If the indices (*i.e.*, the locations) of these *P* largest coefficients of the vector, ***c***, are known, then optimal performance could be achieved: it is possible to exactly recover the magnitude of each of these *P* largest coefficients. This means that it is possible to reconstruct ***c*** (or, equivalently, ***f***) with an accuracy corresponding to its *P* largest coefficients. Now, it can be shown that CS can asymptotically obtain the same accuracy as this optimal solution, as long as the number of random projections is increased by a factor of $\text{O}\left(\log\left(\frac{N}{P}\right)\right)$ [19]. This result implies that compressed sensing's non-adaptive sampling scheme performs as well as a purely adaptive scheme that knows the locations of the *P* largest coefficients. In other words, CS is able to find the *P* largest coefficients without any knowledge of the signal. The only cost is a mild oversampling factor.

## 3. Methods

This section introduces the framework we developed to efficiently compress EEG signals using low energy. A brief overview of the state-of-the-art systems that will be used to compare our results with is also given.

### 3.1. Proposed System

**Figure 2.**
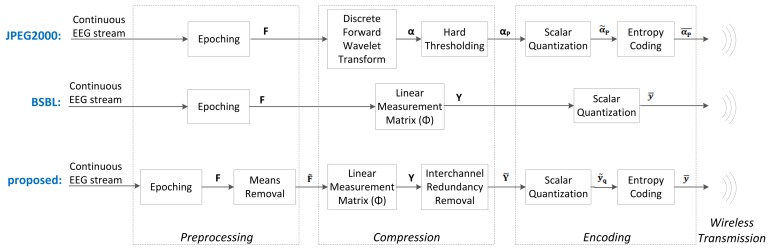
Block diagrams of the sensor node for (1) the JPEG2000-based framework (**Top**); (2) the Block-Sparse Bayesian Learning (BSBL) framework (**Middle**); and (3) the proposed compressed sensing (CS)-based framework (Bottom).

**Figure 3.**
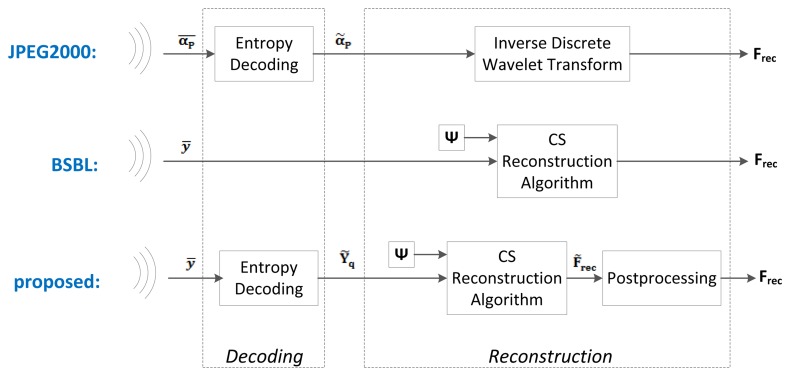
Block diagrams of the server node for (1) the JPEG2000-based framework (**Top**); (2) the BSBL framework (**Middle**); and (3) our CS-based framework (**Bottom**).

Below, we present the different blocks and algorithms that make up our proposed system. We will discuss the preprocessing, the compression, the encoding, the wireless transmission, the decoding and the reconstruction. A block diagram of the proposed system is shown at the bottom of Figures 2 and 3.

#### 3.1.1. Preprocessing

The data is first divided into non-overlapping line segments of length *N*. In our experiments, *N* corresponded to 512 samples for each channel. Note that our framework operates on data from one epoch at a time. Assuming we have ***C*** channels (sensors) of EEG data, after epoching, a total of ***C*** sequences of *N* = 512 data points each are obtained: ***f***_1_, ***f***_2_,…, ***f****_C_*. This forms a matrix, ***F***. Each column of ***F*** contains one of the channels: ***F****_N_*_×_*_C_* = [***f***_1_|***f***_2_| … |***f****_C_*].

The mean of each channel is then removed. The resulting matrix is ***F̃*** = [***f̃***_1_|***f̃***_2_| … |***f̃***_C_]. The means will be added back in the reconstruction phase. Removing the means leads to a higher compression ratio, because the interchannel redundancy removal module (discussed later) performs better on demeaned EEG signals. It also reduces the total range of the signals, which makes it easier to quantize and encode them.

#### 3.1.2. Compression

To compress the de-meaned EEG signals contained in one epoch, we first take their linear random projections and then apply an interchannel redundancy removal module.

##### Measurement Matrix (Φ)

As mentioned in Section 2, the chosen measurement matrix, **Φ**, must be maximally incoherent with the sparsifying dictionary. With a probability asymptotically close to 1, random matrices satisfy this requirement irrespective of the dictionary used. The most often used matrices are random matrices with independent identically distributed entries formed by sampling a Gaussian distribution ( 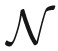 (0,1/*N*)) or a Bernouilli distribution (with $P(\Phi_{i,j}=+1/\sqrt{N})=1/2$, $P(\Phi_{i,j}=-1/\sqrt{N})=1/2)$. In fact, it can be proven that these two types of matrices are optimal. Unfortunately, these matrices are not suitable for WBSN applications. This is because generating a Gaussian random matrix requires a Gaussian random generator at the sensor node (which cannot be efficiently implemented on simple sensor hardware). Moreover, using such a matrix would lead to ***C*** large matrix-vector multiplications for each epoch (one for each of the ***C*** channels). As these operations are energy intensive, they should be avoided in WBSN applications. They are also time consuming and would therefore prevent the system from operating in near real-time mode.

The use of a full Bernouilli matrix would reduce the challenges mentioned above (it is easier to generate its random entries, and it also has simpler multiplication operations), but this unfortunately would still require a high number of computations.

Instead, we use what is known as sparse binary sensing. This was first proposed in [21] and has since been applied to WBSNs ([11,22]). These matrices only contain *d* nonzero entries in each column, and the value of these nonzero entries is 1. The optimal value of *d* (that is, the smallest value for which reconstruction would be stable) is obtained experimentally and is much smaller than *M*. While the theoretical guarantees are not as strong as those for full random matrices, it has been shown that these matrices perform well in many practical applications. There are significant advantages to using such a matrix in WBSNs. The most important one is that the matrix multiplication operation is very simple: in fact, it consists of *Nd* simple additions for each channel.

We propose the use of the same sparse binary sensing measurement matrix for each channel (sensor), so that we can further exploit the interchannel correlations (see the next section). We will test 2 different settings: the first uses a fixed sensing matrix (stored in the sensor node memory) for all epochs, and the second generates a new matrix for each epoch.

We apply the *M* × *N* measurement matrix **Φ** to each EEG channel in order to obtain the compressed measurements: ***Y****_M_*_×_*_C_* = [***y***_1_|***y***_2_| … |***y****_C_*] = [**Φ*f̃***_1_|**Φ*f̃***_2_| … |**Φ*f̃****_C_*].

##### Interchannel Redundancy Removal

Because all EEG channels collect information related to the same physiological signal, there exist large interchannel correlations amongst them. Indeed, channels that are spatially close to one another tend to collect signals that are highly correlated. Because we use the same measurement matrix for each channel, the linear projections of the signals of these channels are also correlated. To remove the redundancies inherent in the multi-channel EEG acquisition, we compute the differences between the compressed values of channel pairs (*i.e.*, between ***y****_i_*, ***y****_j_* for some index pairs (*i,j*) that we carefully select). To select the best channel pairs (*i.e.*, those that will lead to the smallest differences), we run an algorithm at the server node. The server node transmits the channel pairs to the sensor node, which then computes the differences. The redundancy removal algorithm is given in Algorithm 1.


**Algorithm 1**. The interchannel redundancy removal algorithm.
1.Compute ***R***, the matrix of correlation coefficients of the current epoch. Each entry of ***R*** is calculated as follows:
${\text{R}}_{i.j}=\frac{\mathrm{cov}(y_{i},y_{j})}{\sigma (y_{i})\sigma (y_{j})}$  where cov (***a***, ***b***) is the covariance between ***a*** and ***b***, and *σ*(***a***) is the standard deviation of ***a***.2.Subtract the identity matrix ***I*** from ***R*** (because the autocorrelations are not meaningful).3.Define a loop index, *k*, that will be used to track the number of differences taken. Let *k* = 1.4.Find *R*_max_, the maximum absolute value in matrix ***R*** − ***I***. The row and column indices (*i_k_,j_k_*) corresponding to *R*_max_ corresponding to the 2 channels used for the difference. Remove the channel pair (*i_k_,j_k_*) from the correlation pool.5.Check that the selected channel pair is not redundant (*i.e.*, it is linearly independent from the previously selected pairs when we look at it as a system of equations). If it is redundant, discard the current pair. If it is not, keep the current pair and increment *k* by 1.6.Repeat steps 4 and 5 while *k* < *C* or while *R*_max_ < *T*, where *C* is the number of channels and *T* is a user-defined threshold.7.If *k* < *C*, we have run out of good channel pairs and will thus need to pick individual channels (not a difference) to complete our system of equations. Choose a “pair” (*i_k_*,0), such that channel *i_k_* is the channel with the smallest variance that is linearly independent from the previously selected pairs. Increment *k* by 1. Repeat until *k* = *C*.8.Wirelessly transmit the selected channel pairs (*i_k_,j_k_*), *k* = 1 to *C* to the sensor node. These pairs will be used to compute the differences in the next epoch.


The threshold, *T*, controls the minimum acceptable correlation between channel pairs. That is, any channel pair whose correlation falls below *T* will not be selected. If *C* or more pairs are above *T* and are non-redundant, *T* is in fact meaningless. However, if this is not the case, *T* determines the number of differences to be taken. We selected *T* = 0.6, as this was the value that gave us the best results experimentally. Also note that the selected channel pairs are wirelessly sent to the sensor node in a “predictive” manner; of course, the server node does not know what the compressed values of the current epoch are before they are transmitted, and so, the channel pairs are based on the compressed values from previous epochs.

We observed experimentally that for a given dataset, the best channel pairs are mostly stable over time (*i.e.*, the best pairs from one epoch to another are fairly constant). As such, there is no need to repeatedly transmit the best channel pairs. In practice, the best approach might be to periodically update the best channel pairs, in case there has been a shift in the signals.

The sensor node is only responsible for calculating the differences, which is computationally inexpensive. They are calculated for *k* = 1 to *C* as follows:

$${\tilde{\text{y}}}_{k}={\text{y}}_{i_{k}}-\mathrm{sign}(R_{i_{k},j_{k}}){\text{y}}_{j_{k}}$$

After this stage, we obtain a matrix ***Ỹ****_M_*_×_*_C_* = [***ỹ***_1_|***ỹ***_2_| … |***ỹ****_C_*], which contains the difference signals.

**Figure 4.**
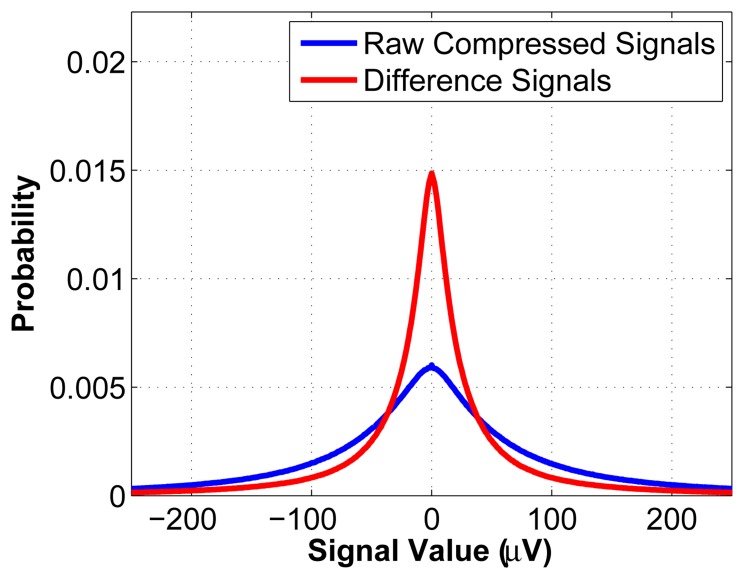
Probability density functions of the raw CS measurements and of the difference signals.

Figure 4 shows the probability density functions (pdf) of the original measurement signals and of the difference signals, taken over 1,000 random windows of a length of 512 of the datasets described at the start of Section 5. As can be seen, the pdf of the difference signals has the desirable property of being narrower than that of the original measurements. This makes it easier to encode the values (it requires fewer bits to do so), which results in a higher compression ratio.

#### 3.1.3. Encoding

After removing the interchannel redundancies, the signals are quantized and an entropy coding scheme is applied. The output of the encoding module is a stream of bits to be transmitted wirelessly.

For quantization, the columns of ***Ỹ****^T^* are first stacked as a vector:

$${\tilde{\text{y}}}_{MC\times 1}=\text{vec}({\tilde{\text{Y}}}^{T})={\left[{\tilde{\text{y}}}_{1}(1){\tilde{\text{y}}}_{2}(1)\ldots {\tilde{\text{y}}}_{C}(1){\tilde{\text{y}}}_{1}(2){\tilde{\text{y}}}_{2}(2)\ldots {\tilde{\text{y}}}_{C}(2)\ldots \ldots {\tilde{\text{y}}}_{1}(M){\tilde{\text{y}}}_{2}(M)\ldots {\tilde{\text{y}}}_{C}(M)\right]}^{T}$$

This type of vectorization ensures that the compressed samples of a single channel are interleaved, *i.e.*, that they do not all come in one burst. In other words, the data is sequenced temporally (by sample) rather than spatially (by channel).

After quantization, the resulting vector is ***ỹ****_q_* = *Q*(***ỹ***), where *Q* is the scalar quantization operator. Assuming that the original signals are represented using 12 bits, we use a 15-bit quantization (to account for the increase in signal range caused by taking the linear projections).

Entropy coding is then applied on ***ỹ****_q_*, as this further reduces the number of bits required to represent the signals. As seen in Figure 4, the distribution of the difference signals, ***ỹ***, is definitely not uniform; in fact, it approximately has the shape of a Gaussian. We exploit this fact to achieve greater compression. Huffman encoding is known to perform well in such cases, so it is chosen as the entropy coding strategy. The codebook is generated offline using the information from Figure 4 and is then stored in memory at the sensor node. We thus obtain a vector ***y̅*** = *H*(***ỹ****_q_*), where *H* is the Huffman encoding operator. Note that for a fixed value for *M*, the length of ***y̅*** varies slightly from one epoch to another, depending on the encoding.

#### 3.1.4. Wireless Transmission

After compression and encoding, the EEG signals are wirelessly transmitted from the sensor node to the server node. As in [11], we assume that each packet has a size of 127 bytes, of which 13 bytes are the media access control (MAC) overhead. Due to the type of vectorization used in the encoding step, each packet contains data from all channels (and not only the data for a single channel or for few channels). Note that we make no further attempt to model the wireless channel and, instead, treat it as a black box.

#### 3.1.5. Decoding

Upon receiving the encoded compressed EEG signals, ***y̅*** (assuming that we are dealing with a perfect channel), the server would first decode them. This is a straightforward decoding operation: ***ỹ****_q_* = *H*^−^*^1^*(***y̅***), where *H*^−1^ is the Huffman decoding operator. We then form an *M* × ***C*** matrix: ***Ỹ****_q_* = [***ỹ****_q_*_1_|***ỹ****_q_*_2_| … |***ỹ****_q__C_*].

#### 3.1.6. Reconstruction

The final step is to reconstruct the original EEG signals from the decoded measurements, ***Ỹ****_q_*. Before applying the CS reconstruction algorithm, we first reverse the effect of the interchannel redundancy module to obtain ***Y****_q_*, the original compressed measurements (before their differences were taken). Once this is done, we can do CS reconstruction. The vast majority of reconstruction algorithms use a dictionary in which the signals are sparse (or at least compressible).

##### Sparsifying Dictionary (Ψ)

As discussed above, one of the main elements of compressed sensing is the selection of a proper sparsifying dictionary, **Ψ**. Different sparsifying dictionaries were tested in [14], but there was no aim at optimizing the chosen dictionaries to obtain the best performance possible (that is, to obtain the dictionary in which EEG signals are the most sparse). Previous work has shown that EEG signals are sparse in the Gabor domain [13].

The Gabor dictionary is a redundant dictionary that provides optimal joint time-frequency resolution [23]. Gabor functions are sinusoidally-modulated Gaussian functions. The atoms in this dictionary are given by:

$$\left[t]g_{i}(n;n_{0},f_{0},s)=K(n_{0},f_{0},s)\cdot \exp\left(-\frac{{\left(n-n_{0}\right)}^{2}}{2s^{2}}\right)\cdot \sin(2\pi \cdot f_{0}(n-n_{0})\right)$$

where *n*_0_ and *f*_0_ are the centers of the Gabor atom, *s* is the spread of the atom and *K*(*n*_0_, *f*_0_, *s*) is a normalizing constant.

We now require a discretization over the *n*_0_, *f*_0_ and *s* parameters; that is, we need to determine how to increment these parameters. For *s*, the chosen discretization is a dyadic scale (base 2). The time increment, *n*_0_, is proportional to the spread, and the frequency increment, *f*_0_, is inversely proportional to the spread. The size of the dictionary depends on the length of the EEG epoch considered in the time domain. To obtain the frequency step and the time step, we rely on the following equations:

$$\begin{array}{cc} \Delta f_{0} & =\frac{\sqrt{8\pi \alpha }}{sN}\\ \Delta n_{0} & =sN\times \sqrt{\frac{2\alpha }{\pi }} \end{array}$$

where *N* is the epoch length and *α* = 0.5ln(0.5(*B* + 1/*B*)). *B* is the base used (2 in our case).

These equations are based on the distance metric for Gabor atoms proposed in [24]. We can show that selecting *n*_0_ and *f*_0_ in this manner provides a dictionary with optimal distance between the atoms.

##### Reconstruction Algorithm

There exists a multitude of reconstruction algorithms for reconstructing the original signals. It is possible to use convex optimization to solve Equation (4). It is also possible to use a greedy algorithm, which looks at finding a suboptimal solution to Equation (3) by iteratively selecting the locally optimal solution, in the hope of getting closer to the globally optimal solution. Such algorithms converge to an acceptable solution faster than convex optimization algorithms. This is, however, at the expense of requiring slightly more measurements.

In our framework, we use a convex optimization algorithm, the Basis Pursuit Denoise algorithm implemented in the SPGL1 package [25]. Based on our tests, this algorithm required fewer measurements (smaller *M*) than greedy algorithms to achieve an equivalent reconstruction accuracy; this results in a higher compression, as a smaller *M* can be used. After performing the reconstruction on each channel data (*i.e.*, on each column of ***Y****_q_*), we obtain the *N* × *C* matrix of reconstructed signals: ***F̃***_rec_ = [***f̃***_1_|***f̃***_2_| … |***f̃****_C_*].

##### Post-Processing

The final step is to add back the means of each EEG channel.

### 3.2. State-of-the-Art Systems

Given that the problem under investigation (EEG compression in a WBSN setting) has only started to be studied recently, there is not a large body of existing literature around it, and identifying a proper benchmark is challenging. EEG compression has been studied quite extensively in the last two decades, but algorithms have not generally been designed for low energy consumption or for implementation on simple hardware. As such, some frameworks can achieve high compression ratios, but they require too many computations or some operations that are too complex given our target sensor hardware, making these frameworks prohibitive in WBSNs. Similarly, WBSNs started to gain momentum in the last decade, but few of them have addressed their applications to EEG signals. As a result, there only exist very few papers that have explicitly studied the problem of EEG compression for WBSNs. In order to identify state-of-the-art systems to compare the performance of our proposed framework, it is therefore necessary to extrapolate the results of this previous research in order to identify schemes that can offer good performance in the context in which we are interested.

We use the following requirements for selecting state-of-the-art systems to compare our system with:

- low energy consumption: the system must not have a high computational requirement at the sensor node in order to conserve energy;
- simple sensor hardware: the system must be simple enough to operate using inexpensive hardware;
- high compression ratio: the achievable compression ratio must be high enough to justify the extra computations carried at the sensor node (as compared to wirelessly sending raw, uncompressed data).

Based on these requirements, we have selected 2 state-of-the-art compression methods to which we compare our proposed framework. These are described in some details below.

#### 3.2.1. JPEG2000-Based Compression

The JPEG2000-based EEG compression scheme was proposed in [26]. Amongst the non-CS methods, it is one of the simplest yet most effective compression schemes. It is a simple wavelet compression algorithm adapted from the JPEG2000 algorithm used for image compression. Its block diagram is shown at the top of Figures 2 and 3. The wavelet coefficients of the raw EEG signals are first computed using the Cohen-Daubechies-Feauveau 9/7 discrete wavelet transform. Depending on the desired compression ratio, a hard threshold is then applied so that only the largest wavelet coefficients of the signal are kept. These large coefficients are then encoded using arithmetic coding. At the server side, the received signals are decoded and then reconstructed by taking the inverse wavelet transform. In our implementation, we used a decomposition level of 7 for the wavelet transform. We determined this level experimentally, as it gave the lowest reconstruction error; furthermore, using more levels did not provide an improvement in reconstruction accuracy.

In contrast to CS schemes, this type of algorithm is adaptive in the sense that it relies on the exact knowledge of the signal to find its largest coefficients. Furthermore, the bulk of the computations is done at the sensor node. We will discuss the implications of these later.

#### 3.2.2. BSBL CS Compression

Block-Sparse Bayesian Learning (BSBL) is a reconstruction method that was proposed in [27] and then applied to EEG signals in [22]. Its block diagram is shown at the middle of Figures 2 and 3.

The main difference between the BSBL framework and our framework is in how the reconstruction is carried. Whereas we assume that EEG signals are sparse in a transform domain (Gabor frames in our case) and use that information to reconstruct the original signals, the BSBL framework does not rely on this assumption. BSBL was first proposed to reconstruct signals that have a block structure, that is, signals that have few blocks containing nonzero entries, and the remainder of the blocks containing only zeros. It was then experimentally shown that the BSBL scheme was effective in reconstructing signals even if their block partition boundaries are unknown or if they do not have any clear block structure. Raw EEG signals fall in this last category.

To carry out the reconstruction, the BSBL framework uses a “sparsifying” matrix, Ψ, which is an inverse discrete cosine transform (DCT) operator (EEG signals are not sparse in the DCT basis, but as mentioned previously, BSBL does not rely on sparsity). We used the bounded-optimization variant of the BSBL family (BSBL-BO) with a block partition of 24 and 20 iterations, as we determined experimentally that these values were the optimal partition size and number of iterations for the best reconstruction accuracy.

## 4. Experimental Setup

We now introduce the experimental setup used to evaluate the selected frameworks. We start by presenting the datasets used, as well as defining the performance metrics selected. We then carry out our experiments to evaluate the choice of the measurement matrix.

### 4.1. Datasets

In order to assess the performance of the different algorithms on a wide range of EEG cases, we selected 3 different databases.

The first one is Dataset 1 of the BCI Competition IV [28]. This dataset was recorded from healthy subjects or generated artificially and contains the data of 7 patients. The recording was made using 59 EEG channels per subject at an initial sampling rate of 1,000 Hz.

The 2 other databases are from Physionet [29]. The first consists of seizure data of 22 pediatric subjects. The recordings contain 21 EEG channels at a sampling rate of 256 Hz [30]. The second database is a collection of 108 polysomnographic recordings of different sleep disorders monitoring. Each recording contains between 5 and 13 EEG channels sampled at 256 Hz [31].

Note that some files in these datasets contain dead channels (*i.e.*, channels where the output is always 0). We removed these channels from our analysis, as they provide no useful information. We also resampled the data to 128 Hz, so as to provide a realistic sampling frequency in the context of telemedicine, as well as ensure uniformity between the datasets. Non-overlapping windows of length *N* = 512 were used in our experiments. Unless specified otherwise, the experiments were carried using 100 randomly extracted windows from each dataset.

The random selection algorithm used is straightforward. Each potential non-overlapping window in the current dataset under study is indexed. The desired number of windows is then drawn randomly, following a uniform distribution. We further ensured that the data used in the experimental setup (mainly for parameter setting) was entirely different from the data used for framework evaluation to avoid in-sample testing.

### 4.2. Performance Metrics

To quantify the compression performance, we used the compression ratio (CR), defined as follows:

(7) $$\text{CR}=\frac{b}{\hat{b}}$$

where *b* is the total number of bits in the original (uncompressed signal) and *b̂* is the total number of bits in the compressed signal.

To test the reconstruction quality, we used the normalized mean square error (NMSE), defined as follows:

(8) $$\text{NMSE}(\text{x},\text{y})=\frac{{\left\|\text{x}-\text{y}\right\|}^{2}}{{\left\|\text{x}-\mu_{x}\right\|}^{2}}$$

where ***x*** is the original vector, ***y*** is the reconstructed vector and *μ_x_* is the mean of ***x***.

The NMSE measures the distance between 2 vectors. Of course, the lower the NMSE, the better the reconstruction. Note that in our formula, we remove the mean of the original signal, so that differences in means between datasets do not bias the results.

### 4.3. Choice of the CS Measurement Matrix

**Figure 5.**
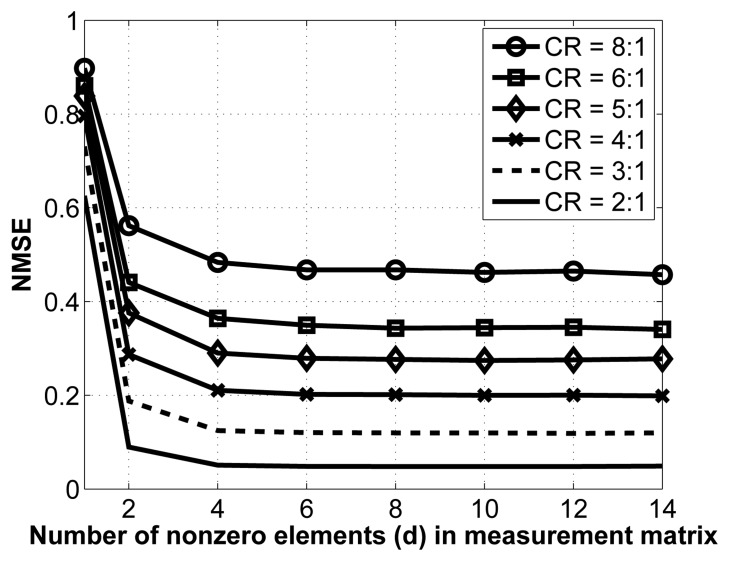
NMSE vs. d for different CRs.

As mentioned in Section 3.1.2., we employ sparse binary sensing, where the measurement matrix, Φ, contains *d* nonzero entries in each column. There are no theoretical guidelines for choosing the optimal value of *d*; we thus determine it experimentally. To do so, we vary the value of *d* and carry out the reconstruction for various compression ratios. Note that for this experiment, we have removed the interchannel redundancy removal and encoding modules to better isolate the effect of changes in the measurement matrix. The results are shown in Figure 5.

As seen from this figure, the NMSE saturates relatively fast, which is a desirable property. Indeed, once the number of nonzero entries in each column, *d*, reaches 8, increasing its value does not yield better reconstruction. As such, we select *d* = 8 in our implementation. Of course, in terms of energy consumption, it is desirable to have the lowest value for *d*, since this results in fewer random linear projections (and, thus, fewer computations) at the sensors.

**Figure 6.**
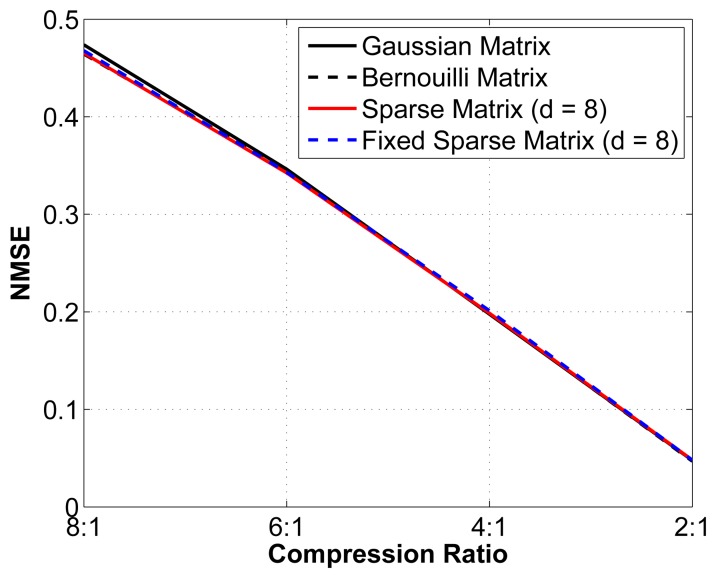
Normalized mean square error (NMSE) *vs*. CR for different measurement matrices.

We then set out to verify the performance of this sub-optimal measurement matrix by comparing the reconstruction performance with that obtained using an optimal random matrix (Gaussian or Bernouilli). We study the reconstruction error for different compression ratios using 4 different matrices: (1) an optimal Gaussian random matrix, in which each entry is formed by sampling an independent and identically distributed (i.i.d.) Gaussian random variable; (2) an optimal Bernouilli random matrix, in which each entry is formed by sampling an i.i.d. Bernouilli random variable; (3) a sparse binary sensing matrix (with *d* = 8) that is different for every epoch analyzed; and (4) a fixed sparse binary sensing matrix with (*d* = 8), *i.e.*, the same matrix is used for all epochs. Again, we have removed the interchannel redundancy removal and encoding modules to better isolate the effect of changes in the measurement matrix. The results are shown in Figure 6.

As seen from Figure 6, although the theoretical guarantees for the sub-optimal sparse binary sensing matrices are weaker than for the optimal Gaussian and Bernouilli matrices, in practice, their performance is statistically almost the same. We can thus safely assume that using a sub-optimal measurement matrix yields near-optimal reconstruction results. Of equal interest is that the fixed sparse binary sensing matrix does not result in degradation in the reconstruction quality. Because the proofs rely on the stochasticity of the matrices used, it is not possible to prove this result theoretically. The use of fixed sparse binary measurement matrices has significant advantages in the context of WBSNs, since we can generate such a matrix offline and then store it in memory at the sensor node. The 2 other alternatives are: (1) generating a new sparse binary sensing matrix at the sensor node for each epoch; or (2) generating the matrix at the server node and then wirelessly transmitting the positions of the nonzero entries to the sensor node. Both alternatives are more energy hungry and are, thus, less desirable.

## 5. Results and Discussion

### 5.1. Energy

We study the energy performance at the sensor node of the two CS schemes (BSBL and the proposed framework) and the JPEG2000 scheme.

After being acquired and digitized, the signals are compressed, encoded and transmitted. The sensing energy is constant for all schemes, since all samples must first be acquired. It was also shown in [32] that the sensing energy is small (about an order of magnitude smaller) compared to the other two classes when using ultra-low-power sensors. As such, we can safely omit it in our analysis. For a fixed compression ratio, the transmission energy is the same for all schemes, because the number of bits to be transmitted would be the same. We thus focus our efforts on the computation energy, which is mainly the energy required for compression and encoding.

We implemented the code in Embedded C and simulated it on a MICAz target platform using the Avrora simulator ([33]). We evaluated the performance based on the total cycle count, the run time and the energy consumption. To estimate the transmission energy, we rely on the experimental work done in [11], in which it was calculated that the transmission of each packet requires 524.72 *μ*J. The results are presented in the top part of Table 1. They refer to one epoch for one EEG channel, so that the difference in the number of channels in the different datasets does not impact the presented results.

As expected, the CS-based schemes significantly outperform JPEG2000 in every category. In fact, even if we use the highest compression ratio for JPEG2000, it will never qualify from an energy perspective when compared with CS. JPEG2000, being an adaptive scheme, relies on a high amount of computations in order to efficiently compress the data while the random projections of CS require a much lower number of computations. It is also interesting to note that small gains (decrease in the number of cycles, run time and energy consumption) are obtained by the proposed framework when the compression ratio increases. This is due to a reduction in redundancy removal and encoding computations. We note that BSBL is slightly more energy efficient than the proposed framework. However, as we will see shortly, that comes at the expense of a worse reconstruction accuracy. We will argue that the small increase in energy consumption is well worth it in this case.

Another interesting consideration would be to look at the comparison between sending compressed and uncompressed data. Sending uncompressed data requires 3.53 mJ of energy per channel (which is the energy required for wireless transmission, as no computations are carried out prior to transmission). We thus note that CS is more advantageous at any compression ratio, whereas JPEG2000 always consumes more energy than even sending uncompressed data.

### 5.2. Reconstruction Performance

The reconstruction performance of the different frameworks over the three datasets is shown at the bottom of Table 1.

In terms of the NMSE, the proposed framework systematically outperforms BSBL. As expected, the reconstruction accuracy of the JPEG2000 framework is generally better than that of the CS-based frameworks, especially at high compression ratios. However, it is interesting to note that when the compression ratio decreases, the gap in reconstruction error quickly shrinks. At lower compression ratios, the proposed framework can even outperform the adaptive JPEG2000 scheme.

We then show that CS-based schemes are more robust to Gaussian noise and packet loss. Such studies have so far been omitted in EEG-based WBSN studies.

#### Gaussian Noise

**Figure 7.**
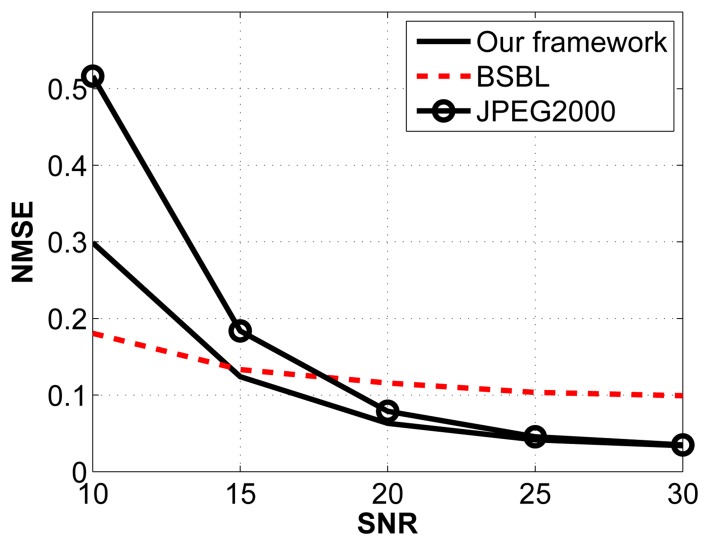
Reconstruction performance under Gaussian noise for a fixed CR (2:1), with varying signal-to-noise ratio (SNR).

For this experiment, we arbitrarily fix the compression ratio to 2:1 and vary the signal-to-noise ratio (SNR) by introducing additive white Gaussian noise with varying standard deviations. The noise frequency is distributed between 0 Hz and 64 Hz. The results are shown in Figure 7.

The JPEG2000 framework is the most affected by Gaussian noise, especially when the SNR is low. Comparing our framework with BSBL, we notice that BSBL performs better at low SNRs, whereas our framework performs better for SNRs higher than 15 dB.

#### Packet Loss

**Figure 8.**
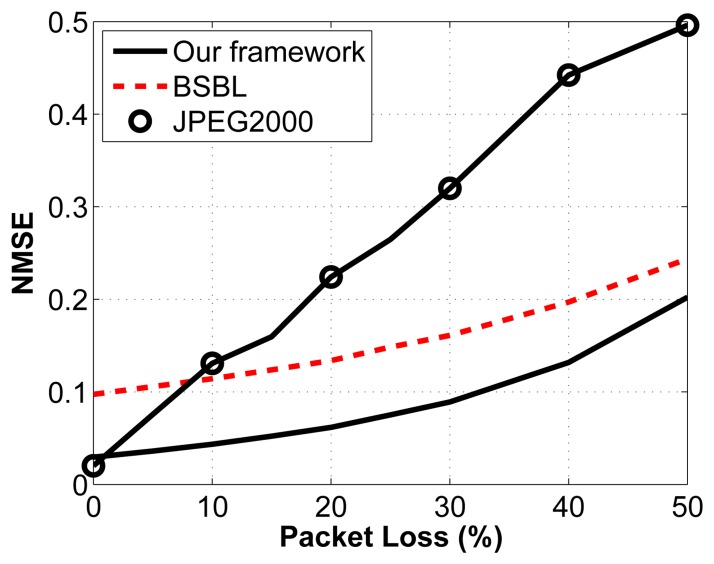
Reconstruction performance under packet loss for a fixed CR (2:1).

We then test the impact of packet loss on the reconstruction accuracy. Again, we arbitrarily select a compression ratio of 2:1. We vary the percentage of packets lost through a noisy channel by randomly choosing the lost packets, which then cannot be used to reconstruct the original signal. We assume that we know how many measurements are contained in each packet and that we know the packet sequence (*i.e.*, we know which packets are dropped in the transmission sequence). For the proposed framework, the packets are built as described in Sections 3.1.3. and 3.1.4. A similar strategy is adopted for the other two frameworks. The results are shown in Figure 8.

The important thing to note about this figure is the relationship between reconstruction accuracy and packet loss. What we notice is that for CS, the slope of the line is much less steep than for the JPEG2000 framework. This property is desirable, since it leads to a more graceful degradation in signal quality. In fact, we can see that as soon as packets are lost, JPEG2000 becomes worse than the proposed framework. Similarly, when the percentage of packets lost go above 9%, BSBL performs better than JPEG2000. Knowing that in WBSN, the packet loss rate can be high, we can see that CS becomes an attractive solution, even from the perspective of reconstruction accuracy. It is also important to understand the possible implications of packet loss for the different frameworks. Because JPEG2000 is adaptive and only sends the largest coefficients, we run the risk of losing the largest coefficients, and we have no control over that: it is simply a matter of luck. In contrast, because the measurements are nonadaptive random projections in the CS frameworks, no measurement bears more weight than another one. In a way, this provides a safety net: even if we have a noisy channel with a high packet loss rate, we know that in all cases, the degradation in signal quality will be proportional to the noise in the channel and will not depend on the timing or location of these losses.

## 6. Conclusions

In this paper, we propose a novel CS framework that exploits both the temporal correlations and the spatial correlations to efficiently compress EEG signals in WBSNs. By providing a simple, nonadaptive compression scheme at the sensor nodes, CS offers a solution to compress EEG signals in WBSNs that is energy efficient, robust to noise and packet loss and results in competitive reconstruction performance as compared to the energy-hungry JPEG2000 compression framework. On the energy front, our proposed CS framework is between five and eight times more energy efficient than the JPEG2000 framework in terms of sensor computations and wireless transmission. We also show that our method achieves a better reconstruction quality than the state-of-the art BSBL method, which also uses CS. This was also the first study in which a wide range of EEG signals are used to validate the performance of different frameworks.

The next steps to demonstrate the applicability of our proposed framework would be to build it in hardware, so as to be able to test its performance and energy consumption under real-life conditions instead of relying on simulations and assumptions. It would also be interesting to look at CS reconstruction algorithms that can directly exploit the spatial correlations by jointly reconstructing the channels. Along the same lines, it may be useful to look at the analysis prior formulation for the CS reconstruction problem, as it could yield an improvement in reconstruction accuracy. Finally, it might be worth looking for an optimal quantization and encoding strategy for CS measurements under a sparse binary measurement matrix. While this problem has been investigated for more traditional CS matrices (see, e.g., [34]), it has not been explored for sparse binary sensing matrices. All of this will be left as future work.

## Figures and Tables

**Table 1.** Energy consumption and reconstruction accuracy for all frameworks. BCI, brain computer interface.

**CR**		**8:1**	**6:1**	**5:1**	**4:1**	**3.5:1**	**3:1**	**2.5:1**	**2:1**

*Energy Consumption*									

**Cycle Count**	**Proposed**	502,200	507,690	512,448	519,402	524,526	531,480	542,757	557,477
	**BSBL**	429,486	429,486	429,486	429,486	429,486	429,486	429,486	429,486
	**JPEG2000**	4,908,731	4,908,367	4,908,055	4,907,535	4,907,171	4,906,703	4,905,975	4,904,831

**Run Time**	**Proposed**	68.11 ms	68.86 ms	69.50 ms	70.45 ms	71.14 ms	72.08 ms	73.61 ms	75.61 ms
	**BSBL**	58.25 ms	58.25 ms	58.25 ms	58.25 ms	58.25 ms	58.25 ms	58.25 ms	58.25 ms
	**JPEG2000**	665.8 ms	665.7 ms	665.7 ms	665.6 ms	665.6 ms	665.5 ms	665.4 ms	665.3 ms

**Computation Energy**	**Proposed**	1.55 mJ	1.56 mJ	1.58 mJ	1.60 mJ	1.61 mJ	1.64 mJ	1.67 mJ	1.72 mJ
	**BSBL**	1.32 mJ	1.32 mJ	1.32 mJ	1.32 mJ	1.32 mJ	1.32 mJ	1.32 mJ	1.32 mJ
	**JPEG2000**	15.11 mJ	15.11 mJ	15.11 mJ	15.11 mJ	15.11 mJ	15.11 mJ	15.10 mJ	15.10 mJ

**Computation + Transmission Energy**	**Proposed**	1.99 mJ	2.15 mJ	2.29 mJ	2.48 mJ	2.62 mJ	2.82 mJ	3.08 mJ	3.49 mJ
	**BSBL**	1.76 mJ	1.91 mJ	2.03 mJ	2.20 mJ	2.33 mJ	2.50 mJ	2.73 mJ	3.09 mJ
	**JPEG2000**	15.55 mJ	15.70 mJ	15.82 mJ	15.99 mJ	16.12 mJ	16.29 mJ	16.51 mJ	16.87 mJ

*Reconstruction Accuracy*									

**NMSE (BCI Dataset)**	**Proposed**	0.3136	0.2171	0.1661	0.1127	0.0856	0.0580	0.0328	0.0108
	**BSBL**	0.5012	0.3723	0.3124	0.2273	0.1910	0.1554	0.1251	0.0919
	**JPEG2000**	0.1159	0.0910	0.0770	0.0612	0.0527	0.0435	0.0333	0.0220

**NMSE (Seizure Dataset)**	**Proposed**	0.6471	0.5049	0.4247	0.3244	0.2746	0.2106	0.1410	0.0743
	**BSBL**	0.6689	0.5381	0.4610	0.3538	0.3076	0.2613	0.2126	0.1597
	**JPEG2000**	0.1967	0.1504	0.1246	0.0971	0.0825	0.0675	0.0517	0.0351

**NMSE (Sleep Dataset)**	**Proposed**	0.3048	0.1754	0.1133	0.0628	0.0430	0.0248	0.0125	0.0036
	**BSBL**	0.4260	0.2983	0.2352	0.1514	0.1189	0.0913	0.0644	0.0404
	**JPEG2000**	0.0568	0.0344	0.0243	0.0156	0.0118	0.0084	0.0055	0.0030
